# Effects of morality and reputation on sharing behaviors in human-robot teams

**DOI:** 10.3389/fpsyg.2023.1280127

**Published:** 2023-12-08

**Authors:** Na Chen, Xueyan Hu, Yanan Zhai

**Affiliations:** School of Economics and Management, Beijing University of Chemical Technology, Beijing, China

**Keywords:** human-robot interaction, morality, reputation, sharing behaviors, reputation concern

## Abstract

**Introduction:**

The relationship between robots and humans is becoming increasingly close and will become an inseparable part of work and life with humans and robots working together. Sharing, which involves distributing goods between individuals and others, involves individuals as potential beneficiaries and the possibility of giving up the interests of others. In human teams, individual sharing behaviors are influenced by morality and reputation. However, the impact on individuals’ sharing behaviors in human-robot collaborative teams remains unclear-individuals may consider morality and reputation differently when sharing with robot or human partners. In this study, three experiments were conducted using the dictator game paradigm, aiming to compare the effects and mechanisms of morality and reputation on sharing behaviors in human and human-robot teams.

**Methods:**

Experiment 1 involving 18 participants was conducted. Experiment 2 involving 74 participants was conducted. Experiment 3 involving 128 participants was conducted.

**Results:**

Experiment 1 validated the differences in human sharing behaviors when the agents were robots and humans. Experiment 2 verifies that moral constraints and reputation constraints affect sharing behaviors in human-robot teams. Experiment 3 further reveals the mechanism of differences in sharing behaviors in human-robot teams, where reputation concern plays a mediating role in the impact of moral constraint on sharing behaviors, and the agent type plays a moderating role in the impact of moral constraint on reputation concern and sharing behaviors.

**Discussion:**

The results of this study contribute to a better understanding of the interaction mechanism of human-robot teams. In the future, the formulation of human-robot collaborative team rules and the setting of interaction environments can consider the potential motivation of human behavior from both morality and reputation perspectives and achieve better work performance.

## 1 Introduction

As the relationship between humans and artificial intelligence robots becomes increasingly intimate, we will no longer see robots only as tools but also as partners and assistants with “life” (Gross et al., 2015). Robots are increasingly collaborating with humans in various situations (Buoncompagni et al., 2018), forming human-robot collaborative teams to complete work tasks together. The role of robots has begun to shift from tools to peer-like teammates capable of assisting and completing joint tasks. This implies that robots may be treated like “humans.” In a human-robot team, people may be willing to share with robot partners and even willing to actively help them (Gaggioli et al., 2021). This tendency in humans is the phenomenon known as anthropomorphism, the perception of humans by assigning mental states, emotions, and intentions to nonhuman agents. Specifically, as long as there are sufficient social clues, people will react to any type of object (such as artificial intelligence robots) in the same way as they do to humans (Hanoch et al., 2021). In human-robot teams, we may see interaction scenarios and behaviors that are somewhat similar to interpersonal communication; that is, humans and robots can work together more naturally and get along harmoniously.

Sharing is seen as a “nonreciprocal pro-social behavior” (Benkler, 2004) and is “the act and process of distributing what is ours to others for their use and/or the act and process of receiving or taking something from others for our use” (Belk, 2007). In human society, sharing is a pervasive and important group behavior that helps us establish and maintain good relationships with others in our daily lives (Dirks et al., 2018). Through the sharing of resources, information, experiences, etc., supportive team interactions can be enhanced, thereby improving team creativity (Gong et al., 2013; Whitehouse et al., 2017; Bastian et al., 2018). Robots exhibit humanoid behaviors such as sharing resources, understanding tasks, and providing assistance, which enable people to naturally interact with robot partners (Chen et al., 2022). The phenomenon of resource sharing similar to that of human teams is also explained in human-robot teams. Sharing can enable humans and robots to complement each other’s abilities, thereby improving team outcomes. At present, research on human sharing behavior mainly focuses on children, who generally seem able and willing to share their resources (Martin et al., 2020). Children’s sharing behavior is not limited to human recipients, but also extends to robots (Martin et al., 2020; Fox and Gambino, 2021). Currently, there is a lack of in-depth investigation on adults’ sharing behavior toward robots.

In human-robot teams, the sharing between humans and robots is asymmetric. On the one hand, due to differences in perception, embodiment, cognitive ability, functional ability, social interaction ability and body, robots and humans function in different ways in teams (Curioni et al., 2019). Robots have a strong ability to gather resources and can share them with humans (Fox and Gambino, 2021). For example, Siri, as a non-physical artificial intelligence product, can provide humans with a large amount of resources, and ChatGTP can even provide solutions to problems for humans. In teams, robots tend to share more resources with humans. On the other hand, based on the concept of the human-centered perspective, the design of robots in human-robot teams is to meet human needs (Kim, 2022), supplement or enhance human abilities, and perform tasks that serve humanity. From the perspective of robot ethics and morality, robots belong to humans, so they can hardly be equivalent to humans in terms of status. Although humans may develop an attachment to or liking for robots, this is a one-way dependency and does not involve shared resources (Fox and Gambino, 2021). Overall, as humanoid partners, robots can be treated like humans, meaning they can share resources. However, due to the different abilities of robots and humans, as well as human-centered theory, in human-robot teams, robots share resources with humans more than humans provide resources to robots. Therefore, it is necessary to explore the differences in sharing behaviors between human-robot teams and human teams.

In human teams, morality and reputation are important factors that affect team sharing behaviors. In terms of morality, understanding and respecting moral principles is one of the necessary conditions for team operation. Morality is conducive to promoting team cooperation, resource allocation, conflict resolution, and mutual benefit and win–win situations (Curry, 2016). In terms of reputation, reputation plays an important role in interpersonal cooperation (Malle et al., 2015). Society expects compliance with social norms, and individuals derive utility from developing a reputation that is consistent with these social norms (Festré, 2010). Attention to reputation can constrain human behavior (Han et al., 2022), and those who share it expect soft benefits such as reputation enhancement and partner recognition as rewards.

In human-robot teams, humans endow robots with unique human attributes, intentions, and emotions. Human-robot interaction is governed by many of the same norms and expectations as human interaction (Edwards et al., 2016). Humans expect robots to follow human social norms, sometimes even more than humans, among which morality and reputation are two important social norms (Sperber and Baumard, 2012). Even humans have a tendency to ascribe morality and reputation to robots. In terms of morality, humans have a tendency to endow robots with morality, especially in emergencies and high-consequence situations, where humans attach greater importance to morality in human-robot interactions (Leonhardt et al., 2011). However, due to the limitations of artificial intelligence and robotics technology, as well as the stage of social cognitive development, the morality attributes assigned to robots are still different from those of humans, and there may also be differences in the moral norms used by humans in their interactions (Seibt et al., 2016). In terms of reputation, there are also differences in the emotional reactions of humans to robot partners and human partners. When confronted with human partners, people exhibit sharing behaviors to ensure their positive reputation or positive feedback. Therefore, our study will explore the differences and motivations of human sharing behavior when facing different types of agents.

As an important component of future collaborative teams, robots are endowed with anthropomorphic attributes and treated like humans. Humans will assign social rules including morality and reputation to human-robot teams. However, due to the degree of anthropomorphism of robots and the concept of Human-Centered Perspective, there may be differences in the social rules used in human-robot teams compared to human teams, so the sharing behaviors of humans when facing robot partners may be different from when facing human partners. Therefore, this study aims to explore the differences in sharing behaviors between human and human-robot teams, as well as the impact mechanisms of morality and reputation.

### 1.1 Sharing behaviors

Sharing, which involves distributing goods between individuals and others, involves individuals as potential beneficiaries and the possibility of giving up the interests of others (Paulus, 2014). Resource sharing is a subtype of giving in which some portion of a set of resources is allocated to another individual. The sharing of resources represents a willingness to sacrifice personal gains out of concerns for fairness, equality, and the needs of others (Ongley and Malti, 2014). Sharing may provide reciprocal benefits, which is a manifestation of caring for others, even at the cost of sacrificing oneself (Ongley and Malti, 2014). Therefore, it is considered a typical indicator of pro-social behavior and willingness to comply with social norms (House and Tomasello, 2018). Due to the existence of sharing, human society can develop better (Matsumoto and Juang, 2016).

Research on human-robot interaction is often based on theories and conclusions related to interpersonal interaction. At present, research on the influencing factors of sharing behaviors in human-robot teams mainly explores aspects such as sharing the characteristics of both parties, sharing the relationship between both parties, and experimental paradigms. In terms of sharing the characteristics of both parties, research has found that humans have a tendency to anthropomorphize robots and assist them. They are influenced by robots in decision-making and share their expensive resources with robots. With robots endowed with emotional states, humans will share more resources (Nijssen et al., 2021a). In terms of sharing the relationship between the two parties, people tend to respond more positively to robots within the group than those outside the group, believing that robots within the group are more humanoid, warmer, and willing to share more resources (Eyssel and Kuchenbrandt, 2012). In terms of experimental paradigms, Nijssen et al. (2021a) found that people are more able to allocate resources equally in a noncostly resource allocation task by comparing the mini dictator experiment (where dictators decide whether to share some of their own resources) with a resource allocation task (where dictators decide whether to share resources that do not belong to them). At present, there are almost no articles systematically exploring sharing behaviors in human-robot teams from the perspective of social norms. This article explores the impact of two typical social norms, morality and reputation, on sharing behaviors in human-robot teams.

In the current study, it is not yet clear whether the degree of sharing with human robot companions is consistent. Some studies suggest that humans treat humans and robot partners equally. Torta et al. (2013) reported that rejection scores in the ultimatum game are higher in the case of a computer opponent than in the case of a human or robotic opponent, indicating that people might treat a robot as a reciprocal partner. De Kleijn et al. (2019) used the ultimatum game to indicate that participants shared an equal proportion of the amount with all types of opponents. There are also studies that suggest that humans tend to share more with human partners compared to robot partners. Through ultimatum games, it has been found that the money provided to robots is significantly less than the money given to human agents (Terada and Takeuchi, 2017; Hsieh et al., 2023). Therefore, this article will explore the differences and motivations of human sharing behavior when facing different types of agents.

At present, research on sharing behaviors mainly adopts methods derived from economic game theory, especially the dictator game experimental paradigm (Karayiannis and Hatzis, 2012). Dictator games are widely regarded as a measure of sharing, a mature (van Dillen et al., 2021), simple, and externally effective method to measure sharing behaviors. They allow for systematic comparison of sharing behaviors among different individuals and backgrounds, with selfless allocations of resources having no external benefits. The shared-with person has no right to refuse the offer, and selfish sharing has no external consequences (Ongley and Malti, 2014). In the simplest case of the dictator experiment, two players anonymously participate in the experiment. One player, as a dictator (i.e., sharer), proposes how to allocate (or not allocate) a given amount to the other player. The other player, as the responder (i.e., the one being shared with), can only accept a quote. Sharing is done anonymously, and the recipient has no opportunity to respond, retaliate, or evaluate the dictator (Gummerum et al., 2010). In the dictator experiment, the behavior of a dictator is influenced not only by maximizing their own financial gain but also by social norms, which constrain self-interested behavior by considering the social impact of their own behavior (Gummerum et al., 2008). The current research on human-robot interaction mainly uses dictator or ultimatum experimental paradigms to explore human acceptance and refusal behavior toward robot quotes (De Kleijn et al., 2019).

### 1.2 Morality in human-robot interaction

In human society, morality plays an important role in influencing various human behaviors. Understanding and respecting morality is a condition for team operation, and team members’ adherence to ethics effectively promotes overall team cooperation (De Groot and Steg, 2009). Morality includes both willingness to engage in beneficial behavior and avoidance of harmful behavior that affects the interests of others. In a team, members’ behavior must follow ethical rules, and any unethical behavior is considered shameful (Karayiannis and Hatzis, 2012). Morality improves the ability of individuals to regulate behavior in social relations (Ogunfowora et al., 2021). Under moral considerations, people consider their own actions to avoid harming the interests of others.

In human-robot teams, people apply the same concepts, processes, and warrants when making moral judgments about humans and robots as in human teams (Rosenthal-von der Pütten et al., 2013), but there are still significant differences. On the one hand, when individuals believe that robots can feel or look more like humans, they will pay more moral attention to robots (Nijssen et al., 2019). However, due to the humanoid level of robot appearance and behavior, people may apply different moral norms to humans and robots (Voiklis et al., 2016; Giroux et al., 2022). Even when robots exhibit the same behavior as humans, there may be differences in people’s moral judgments about such behavior. For example, compared to humans, robots exhibiting utilitarian behavior in moral paradoxes (such as the decision to sacrifice one person to save four people) are considered more acceptable. In contrast, if robots do not engage in such utilitarian behavior, people often consider their behavior to be immoral (Nijssen et al., 2021b); this tendency to make moral judgments about interacting objects is inconsistent or even opposite to that in human society. On the other hand, when faced with robot partners and human partners, there may also be differences in the moral behavior of human individuals. Study found that in regard to the interests of both human and robot partners, people only consider the needs of human partners and do not consider the needs of robot partners when making choices (Voiklis et al., 2016). If a person behaves the same way toward a robot as humans, people might perceive those decisions as unsettling acts that lack “something” inherent in the moral decisions that are made by humans (Kahn et al., 2012).

Any sharing behaviors within a team are influenced by morality, which affects the resource allocation of individuals as sharers between two or more parties (Strauß and Bondü, 2023). Malle and Scheutz summarized the ethics that people should follow when sharing, one of which is to prohibit behaviors that harm others for their own benefit (Malle and Scheutz, 2019). Research has shown that even if the behavior they engage in conflicts with their personal desires, people still act according to morality (Gummerum et al., 2010), which means that individuals are influenced by morality and exhibit more resource-sharing behaviors while reducing selfish behavior. Research using the dictator experimental paradigm has shown a strong relationship between morality and individual sharing behaviors (Schier et al., 2016). Most behavior of sharing money is for moral reasons, and even if it is not in one’s own interest, people tend to appear fair through sharing (Ongley and Malti, 2014).

Previous studies have shown that personification theory may be a mechanism by which individuals apply different moral norms to human and robot partners in team sharing, exhibiting different sharing behaviors. In other words, even if robots are anthropomorphized, when confronted with both humans and robots at the same time, it is difficult for human individuals to put them in an equal position, and they do not have the same instinctive reaction to robots as they do to other humans (Rosenthal-von der Pütten et al., 2013), so they show different sharing behaviors.

### 1.3 Reputation in human-robot interaction

In human society, reputation plays an important role in the cooperation between people (Malle et al., 2015). The reciprocity of reputation explains that people engage in sharing behaviors to ensure their own good reputation (Michael and Salice, 2017), which means that the motivation for people to engage in sharing behaviors may be to gain recognition from others, thereby helping them gain a good reputation and indirectly increasing the chances of receiving help in the future when needed (Shi et al., 2010). Human behavior is strongly influenced by the presence of others, and obtaining a good reputation or avoiding a bad reputation is a powerful motivation for human behavior. When people expect their reputation to be spread to others, they are found to act in a more altruistic way (Mifune et al., 2010). People’s concern for their reputation involves thinking about how others perceive them. Those who share are considered generous, while those who do not share are considered greedy (Izuma, 2012), which means that human behavior needs to meet the interests and expectations of others to a certain extent. In economic games, when anonymity is guaranteed, people tend to act in a more egoistic manner, while when anonymity is not guaranteed, people show more pro-social tendencies and share more resources (Sperber and Baumard, 2012).

Reputation affects sharing behaviors within a team (Hashimoto et al., 2014). People not only hope to maximize their own utility when sharing but also consider sharing with others due to their reputation (Kogut, 2012), which may have an impact on sharing behaviors. People exhibit more sharing behaviors to ensure that they gain a good reputation or positive feedback (De Cremer and Mulder, 2007). Research has shown a positive correlation between reputation and the amount received by the one being shared with (Conte, 2022). Reputation can also serve as a control over selfish behavior. In economic games, sharers learn to behave more selflessly when their reputation is threatened (Benenson et al., 2007); that is, due to the influence of reputation factors, they control their selfish behavior and demonstrate sharing behaviors with their partners.

In human-robot teams, due to the anthropomorphic appearance and behavior of robots, humans may perceive them as living, perceptual, and conscious anthropomorphic entities and attribute psychological characteristics to them (Balle, 2022). Michael and Salice (2017) found that people apply the same psychological mechanisms when thinking about and evaluating robot behavior as when thinking about and evaluating human behavior. From this perspective, when sharing with robots, people may also consider their how they are perceived from the robots’ perspective and take actions to maintain their reputation. However, there has been no in-depth exploration of whether people’s consideration of reputation when sharing with robots is consistent with that of human teams.

Current research suggests that personification theory and reputation reciprocity can jointly explain the different sharing behaviors of individuals toward human and robot partners in team sharing (Shi et al., 2010). Even if robots are anthropomorphized, it is difficult for humans to naturally expect to establish long-term reciprocal relationships with robots when confronted with both humans and robots. Therefore, they will not gain recognition from robots for their sharing behaviors and have a strong expectation of obtaining more cooperation and sharing from robots in the future, that is, humans will show different sharing behaviors when confronted with human and robot partners.

### 1.4 The present study

When robots have the autonomous ability to integrate into human society, they can form teams with humans and become partners to complete tasks together (De Cremer and Mulder, 2007). In human-robot teams, robots exhibit anthropomorphic behaviors such as understanding tasks, sharing resources, and providing assistance. These anthropomorphic behaviors enable humans to naturally interact with robot partners and make humans willing to share with robot partners. Even though humans and robots can interact more like human teams, in human-robot teams, it is still difficult for humans to place their human and robot partners in the same position, and there is no instinctive response to robots like there is to humans (Rosenthal-von der Pütten et al., 2013), which means that there may be differences in sharing behaviors when confronted with robot and human partners, and there are differences in the constraints of morality and reputation on sharing behaviors between human-robot teams and human teams. To deeply and systematically explore this question, this study adopts the dictator game experimental paradigm to conduct three experiments in sequence. The first experiment aims to explore whether there are differences in sharing behaviors between human-robot teams and human teams. The second experiment further explores whether sharing behaviors in human-robot teams are influenced by moral constraints and reputation constraints. Finally, the third experiment aims to reveal the mechanisms by which the sharing behaviors of human-robot teams are influenced by morality and reputation.

### 1.5 Purpose and hypotheses

Robots and humans have inevitably come together as teams to complete tasks and share resources (Correia et al., 2019). The appearance and behavior of robots can be very similar to humans, and we treat robots as if they possess human attributes, including morality (Coeckelbergh, 2014). The process of human-robot interaction is partially dominated by interpersonal norms and expectations (Banks, 2021). Humans attribute emotions and intentions to robots (Coeckelbergh, 2014), but the social norms imposed on robots and humans may be different; that is, humans may not be able to treat robot partners and human partners equally. It is currently unclear whether human sharing behaviors toward robot partners differs from those toward human partners. Therefore, Experiment 1 will explore this question, Hypothesis 1 is proposed:

*H1*: There is a difference in human sharing behaviors when the agents are robots and humans.

In human teams, social norms influence sharing behaviors, and morality and reputation are two important foundations. Morality influences team behavior, and any sharing behaviors are considered to follow moral rules. Compared to machine decision-making, people often make economically more unfavorable decisions because they are influenced by morality when making decisions. Individuals consider ethical norms when sharing resources with human partners, exhibit more sharing behaviors, and reduce selfish behavior, even if it contradicts their personal desires (Gummerum et al., 2010). However, it is not yet certain whether moral constraints equally affect the behavior of sharing with robot partners.

In addition, reputation plays an important role in interpersonal cooperation (Malle et al., 2015). For the sake of a good reputation, people act in a more altruistic way (Piazza and Bering, 2008) or learn to behave more selflessly when their reputation is threatened (Benenson et al., 2007). The focus on reputation in human teams promotes sharing behaviors. However, it is not yet certain whether reputation constraints equally affect the behavior of sharing with robot partners.

The experiment 2 further explores the impact of moral constraints and reputation constraints on sharing behaviors in human-robot teams. Therefore, Hypothesis 2 is proposed:

*H2*: When the agents are robots and humans, moral constraints and reputation constraints have different impacts on sharing behaviors.

Human-robot interaction is governed by many social norms and expectations similar to interpersonal interaction (Edwards et al., 2016), but the impact is not entirely consistent (Malle et al., 2015; Voiklis et al., 2016; Giroux et al., 2022). Compared to sharing with humans, when sharing with robots, humans believe that they have less moral responsibility for each other and attach less importance to their reputation. Krebs et al. believe that the purpose of moral behavior is to help individuals gain a good reputation (Sperber and Baumard, 2012). Trivers proposed that behavior based on moral orientation is more conducive to ensuring a good reputation than directly pursuing reputation (Sperber and Baumard, 2012). When there are differences in sharing behaviors, on the one hand, it may only be due to moral reasons that different sharing decisions are made, and on the other hand, it may be because people’s sharing behavior is due to considerations of their own reputation impact. However, current research has not yet explored these two potential mechanisms in depth. The aim of the experiment 3 is to further reveal the impact mechanism of moral constraints and reputation concerns.

Research has shown that morality has a certain impact on reputation (Sperber and Baumard, 2012; Torta et al., 2013; Schier et al., 2016). Ensuring a good reputation is one of the functions of morality, and the pursuit of reputation itself is an adaptation to morality (Nordin, 2015). Applying moral pressure can strengthen the importance of reputation, which means seeking respect for oneself and aligning one’s behavior with the requirements of morality. Morality can also be seen as a self-interest-oriented problem; that is, as the role of morality increases the importance of reputation, people are more likely to give up short-term gains (Sperber and Baumard, 2012) and share more resources.

The degree of importance attached to reputation, also known as reputation concern, can serve as a control over selfish behavior. The more people value reputation, the more they perceive the impact of reputation in sharing decisions, and the more they exhibit sharing behaviors. People are highly sensitive to reputation threats (Kupfer and Giner-Sorolla, 2021), and when they perceive the consequences of reputation, they are prone to adjusting their behavior, such as being more generous, willing to cooperate, and exhibiting more moral behavior in front of observers (Altay et al., 2020; Kupfer and Giner-Sorolla, 2021). A number of game theory experimental results show that sharing in the economy is typically guided by reputation concerns (Sperber and Baumard, 2012). When it is hinted to participants that their behavior will be noticed by other agents, even if they are anonymous to each other, and they realize that some of their behaviors may be annoying, that is, when their reputation is negatively affected, these participants will behave more generously and share more resources. Further research has shown that even if participants and followers are anonymous to each other, receiving social signals of reputation being threatened can make scorers more generous (Capraro et al., 2021).

Establishing and maintaining a person’s reputation is one of the functions of morality. The pursuit of reputation itself is an adaptation to morality (Nordin, 2015), and moral constraints affect reputation concerns (Sperber and Baumard, 2012; Torta et al., 2013; Schier et al., 2016). Attention to reputation can promote sharing behaviors in economic games (Dana et al., 2006), where people exhibit more sharing behaviors. Therefore, Hypothesis 3 is proposed:

*H3*: Reputation concern plays a mediating role between moral constraints and sharing behaviors.

**Figure 1:**
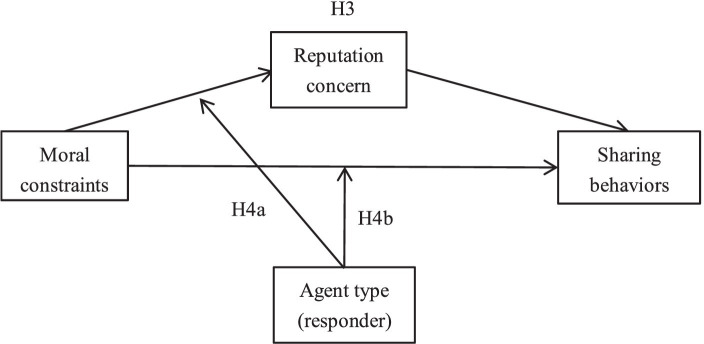
Research theoretical conceptual model.

In interpersonal and human-robot teams, the moral obligation used may differ, and there may be differences in the guarantee of reputation; that is, people can perceive differences in the reputation they gain by adhering to morality. In human teams, even if moral constraints are not emphasized, moral rules will naturally be applied, and people’s attention to morality is related to their concern for reputation. In human-robot teams, the impact of moral constraints and reputation concerns is not spontaneous. Emphasizing moral constraints increases the perceived importance of reputation when humans interact with robots (Figure 1). Therefore, Hypothesis H4a is proposed:

*H4a*: Agent type plays a moderating role in the impact of moral constraints on reputation concerns.

Sharing behaviors within the team are influenced by morality. When not emphasizing moral constraints, there are differences in the morality applied to human and robot partners, and there are also differences in their sharing behaviors. Even though humans have a tendency to impose moral values on robot partners, their perception of them is not equivalent to their perception of human partners (Sommer et al., 2019); that is, in human-robot teams, humans rarely use morality to constrain their own behavior. When emphasizing moral constraints, robot partners are given a certain degree of moral attention (Seibt et al., 2016), leading to changes in human sharing behaviors toward them. Therefore, Hypothesis 4b is proposed.

*H4b*: The agent type plays a moderating role in the impact of moral constraints on sharing behaviors.

## 2 Experiment 1

### 2.1 Experimental design

#### 2.1.1 Participants

This experiment recruited 18 participants, including 12 women and 6 men, with an average age of 20.56 years old (SD = 2.22). They were undergraduates and graduate students in China.

#### 2.1.2 Task and experimental design

The study adopts the dictator game experimental paradigm. In the lab experiment, participants played the role of dictators (i.e., sharers) and completed the experiment together with an anonymous fake participant (human or robot) who played the role of responders (i.e., the one being shared with). The participants were instructed to imagine receiving a donation of 100 yuan. The amount allocated by the participants is fictitious, unexpected and not earned by themselves. The participants need to allocate the 100 yuan donation between themselves and the responders-up to giving all the money to the responders without any profit, and at the least not giving it to the responders, and retaining all the money themselves. Responders can only passively accept the allocation plan provided by the participants and cannot take any action.

In the lab experiment, each participant participated in the experiment separately and completed the task together with the fake participant using a computer. The experimental task consists of two rounds of independent dictator task trials, with one through a human-robot team and the other going through a human-human team. In the human-robot team, the fake participant who plays the responder is a robot, while in the human-human team, the fake participant who plays the responder is a human. The order of responder type (humans or robots) experienced by different participants is random. After the experimental design was completed, we recruited 10 students for a pre-study and watched a 10 s video of human and robot responders. In the video, the human and robot responders performed several simple actions, such as smiling and raising their hands. After the participants watched a 10 s video, a post event interview was conducted, which mainly included whether robots and humans could be effectively distinguished, whether the robot’s identity was recognized, and suggestions for relevant scenarios. According to the interview results, most participants were able to distinguish between the robot and the human responder within a valid time frame, and all students believed that they could accept the identity of the robot. Some participants raised the issue of unclear video quality.

**Figure 2:**
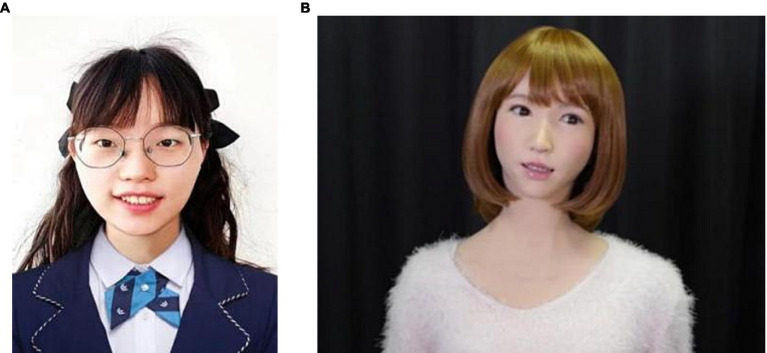
The personal information of the human/robot responder. **(A)** Human responder image (Female, 24 years old, graduate student in progress). **(B)** Robot responder image (Female, 24 years old, graduate student in progress).

The experiment includes an independent variable, agent (within-group variable, including two levels: human or robot), and a dependent variable, sharing behaviors. Among them, the independent variable, agent, is controlled by asking participants to watch a personal information profile of the type of participant (human or robot) and a 10 s image video at the beginning of the experimental task. To ensure that the participants were correctly aware of the type of responder, we used both image and video methods. Specifically, we first showed the participants a 10 s dynamic video of responders (robot or human), in which the responders performed several simple actions, such as smiling and raising their hands. After watching the video, the participants needed to select the object just displayed from 10 static images (either human or robot), and the subjects who selected correctly could participate in subsequent trials. The agent setting method for this variable is referred to as the 10-s video and 10-picture selection method. An example of the personal information of the participants displayed in the trial is shown in Figure 2.

#### 2.1.3 Measures

The dependent variable, sharing behaviors, is measured by the amount of money shared by participants with responders in the trial of the dictator experimental paradigm.

In addition, the experiment measures participants’ self-reported risk preference and perceived intelligence as control variables. The risk preference scale developed by Weber et al. (2002) was used, which includes a total of 40 items. The Cronbach’s alpha is 0.87, and the typical title is “Betting a day’s income at a high stack poker game.” Perceived intelligence was measured using the intelligence scale from the Godspeed questionnaire (Bartneck et al., 2009), which had five items. This study did not involve measuring robot abilities, so the questionnaire item “Incompetent/Component” was deleted, and the final perceptual intelligence scale used consisted of four questions. The two questionnaires were measured with a five-point Likert scale, and the subjects were required to score the possibility of their participation in the activities or behaviors described in the questions, 1 = strongly disagree; 5 = strongly agree.

#### 2.1.4 Procedure

The experiment lasted approximately 10 min in total. Before the trial began, participants signed an informed consent form, read the experiment instructions, and filled out a pre-experiment questionnaire, including gender, age, and risk preference. Then, the participants completed two rounds of dictator decision-making trials with human and robot agents and watched the participants’ information and videos before each round of trials began. Finally, after two rounds of trials, the participants filled out a post-experiment questionnaire, including the perceived intelligence scale. The research was approved by the ethics committee of the university where it is located.

### 2.2 Data analysis and results

According to the linear regression results, the participants shared numerically more money with human responders than with robot responders (*F* = 6.067, *p* = 0.019, *d* = 0.845; for humans: *M* = 27.5, SD = 21.49; for robots: *M* = 10.61, SD = 18.37). This study answers hypothesis 1, which states that human sharing behaviors exhibit significant differences under different sharing agent conditions.

## 3 Experiment 2

### 3.1 Experimental design

#### 3.1.1 Participants

This experiment recruited a total of 74 participants, including 34 women and 40 men, with an average age of 23.23 years old (SD = 1.28). The subjects were randomly divided into four groups: the morality group (18 people), the immorality group (18 people), the reputation group (20 people), and the regardless-of-reputation group (18 people). There was no significant difference in the sex ratio or age among the four groups (both *p* > 0.05).

#### 3.1.2 Task and experimental design

Experiment 2 adopts the same dictator experimental paradigm as Experiment 1. The experimental task involves two independent rounds of dictator decision-making trials, with one through a human-robot team and the other through a human-human team. In the human-robot team, the fake participant who plays the responder is a robot, while in the human-human team, the fake participant who plays the responder is a human. The order of agent type (humans, robots) experienced by different participants is random. The difference from the experimental mechanism of Experiment 1 is that Experiment 2 added pre-experimental settings to control the two levels of moral constraints and reputation constraints on the independent variables. The experiment consists of two independent variables: moral constraints (between-group variables, including two levels: morality and immorality) and reputation constraints (between-group variables, including two levels: reputation and regardless-of-reputation), a moderating variable of agent type (within-group variables, including two levels: robot and human), and a dependent variable of sharing behaviors.

Among them, the independent variable moral constraint is controlled by whether the participant is informed before the experiment that their sharing behaviors will cause the participant’s human agent to go bankrupt or the robot agent to be scrapped. For the morality group, before the trial, participants were not only told to watch the information and videos of the one being shared with but also told that if the amount shared was too small when sharing with human agents, the other party would have no money and their lives would become even more difficult. When sharing with the robot agent, too little sharing would result in the robot being penniless and unable to receive subsequent maintenance, and the robot would be scrapped. The purpose of such experimental settings is to make participants feel stressed or have a sense of guilt and moral perception. For the immorality group, Experiment 2 does not add similar experimental settings, and the experimental conditions are the same as those of Experiment 1, which does not emphasize moral considerations.

The independent variable reputation constraint is controlled by whether participants are informed before the experiment that their sharing behaviors will be publicly evaluated. For the reputation group, before the experiment, participants were not only told to watch the information and videos of the sharing subjects but also informed that the one being shared with (humans or robots) would publicly evaluate the results they shared to draw attention to their own reputation. For the regardless-of-reputation group, Experiment 2 does not add similar experimental settings, and the experimental conditions are the same as those of Experiment 1, which does not emphasize reputation considerations.

#### 3.1.3 Measures

To determine the morality/reputation-related experimental settings that each group of participants truly trialed, that is, whether the participants correctly perceived morality/reputation constraints, after observing the responder information and being informed of the experimental settings of their group, the participants were required to fill out the perceived moral scale (for both the morality group and the immorality group) or the perceived reputation scale (for both the reputation group and the regardless-of-reputation group). Perceived morality uses the Moral Foundations Questionnaire to measure the perception of whether there are moral rules in the situation. The scale contains a total of 30 items, with Cronbach’s alpha of 0.88 and a typical title of “Whether or not someone acted unfairly.” The perceived reputation scale adopts the reputation scale developed by Zinko et al. (2016) to measure the perception of whether reputation is considered in the current situation. The original scale expressions are suitable for reputation evaluation of specific objects. Therefore, this experiment changed the statement of the original scale to personal reputation evaluation for specific situations, such as changing “People believe in this person” to “In this situation, partners trust my decisions very much.” The scale contains a total of 19 items, with a Cronbach’s alpha of 0.98. Both scales were measured using a 5-point Likert scale, with 1 = Strongly Agree and 5 = Strongly Disagree.

The dependent variable, sharing behaviors, is measured by the amount of money shared by participants with responders in the trial of the dictator experimental paradigm.

The variable agent type is controlled using the 10-s video and 10-picture selection method.

In addition, the experiment measures participants’ self-reported risk preference and perceived intelligence as control variables. The questionnaire and measurement method used are consistent with Experiment 1.

#### 3.1.4 Procedure

The experiment lasted approximately 15 min in total. Before the experiment began, the participants signed an informed consent form, read the experiment instructions, and completed the pre-experiment questionnaire, including gender, age, and risk preference. Then, the participants completed two rounds of dictator decision-making trials where the responder was a human or a robot. Before each round of trials, participants were required to complete a 10-s video and 10-picture selection, set up a morality constraint or reputation constraint experiment for the perceived group, and fill out a perceived moral or reputation questionnaire. After two rounds of trials, the participants filled out the post-experiment questionnaire, including the Perceived Intelligence Scale. The research was approved by the ethics committee of the university where it is located.

### 3.2 Data analysis and results

According to the independent sample *t* test results, for perceived morality, the morality group had significantly higher perceived morality compared to the immorality group when confronted with both human and robot agents (both *p* < 0.001). For perceived reputation, whether confronted with human agents or robot agents, the perceived reputation of the reputation group is significantly higher than that of the regardless-of-reputation group (both *p* < 0.001). This indicates that the experimental setup is effective, and each group of participants correctly perceived the level of morality/reputation of the experimental setup in their group, allowing for further data analysis. The specific data analysis results are shown in Table 1.

**Table 1:** Perceived morality/reputation *t* test results for four groups.

	Perceived morality				Perceived reputation			
	Morality group	Immorality group	*t*	*p*	Reputation group	Regardless-of-reputation group	*t*	*p*
Human agent	3.92 (0.34)	3.63 (0.38)	2.625	< 0.001	3.93 (0.39)	3.53 (0.32)	2.735	< 0.001
Robot agent	3.66 (0.47)	2.72 (0.41)	1.363	< 0.001	3.65 (0.36)	2.92 (0.40)	1.521	< 0.001

*The standard deviation is shown in parentheses.

The impact of moral constraints on sharing behaviors was analyzed. Comparing the morality group and the immorality group, according to the linear regression results, the interaction term between moral constraint and agent type has a significant impact on sharing behaviors (*F* = 7.062, *p* = 0.010), indicating that when the participants are of different types, the impact of moral constraints on sharing behaviors is significantly different. The posttest results showed that in human teams, emphasizing moral constraints had no significant impact on sharing behaviors (*F* = 0.214, *p* = 0.647, *d* = 0.119; the morality group: *M* = 32.26, SD = 14.47; the immorality group: *M* = 30.49, SD = 15.25). In human-robot teams, emphasizing moral constraints had a significant impact on sharing behaviors (*F* = 29.414, *p* < 0.001, *d* = 1.286; morality group: *M* = 34.38, SD = 18.68; immorality group: *M* = 10.61, SD = 18.27). This indicates that when confronted with human agents, there is no need to deliberately emphasize that the participants’ sharing behaviors will naturally consider morality and adjust their own behavior according to the requirements of moral rules. When in human-robot teams, sharers could not automatically consider moral implications. It is necessary to deliberately emphasize the existence of morality so that participants can constrain their self-interest behavior and exhibit certain sharing behaviors under the constraints of morality. The specific data analysis results are shown in Table 2.

**Table 2:** Results on the impact of morality constraints and reputation constraint on sharing behaviors.

	Moral constraint				Reputation constraint			
	Morality group	Immorality group	*F*	*p*	Reputation group	Regardless-of-reputation group	*F*	*p*
Human agent	32.26 (14.47)	30.49 (15.25)	0.214	0.647	32.77 (19.64)	31.24 (14.79)	0.362	0.552
Robot agent	34.38 (18.68)	10.61 (18.27)	29.414	< 0.001	34.45 (16.24)	12.34 (15.56)	10.862	0.002

^*^The standard deviation is shown in parentheses.

The impact of reputation constraints on sharing behaviors was analyzed. Compared with the reputation group and the regardless-of-reputation group, according to the linear regression results, the interaction term between reputation constraint and agent type has a marginally significant impact on sharing behaviors (*F* = 3.981, *p* = 0.050), indicating that when the participants are of different types, the impact of reputation constraints on sharing behaviors is significantly different. According to the posttest results, emphasizing reputation constraints has no significant impact on sharing behaviors in human teams (*F* = 0.362, *p* = 0.552, *d* = 0.088; the reputation group: *M* = 32.77, SD = 19.64; the regardless-of-reputation group: *M* = 31.24, SD = 14.79). In human-robot teams, emphasizing reputation constraints has a significant impact on sharing behaviors (*F* = 10.862, *p* = 0.002, *d* = 1.390; the reputation group: *M* = 34.45, SD = 16.24; the regardless-of-reputation group: *M* = 12.34, SD = 15.56). This indicates that in human teams, there is no need to emphasize that participants will autonomously consider the series of reputational impacts generated by their own behavior and adjust their behavior accordingly. In human-robot teams, if the potential impact of reputation is not emphasized, participants will not actively adjust their behavior based on the impact of reputation. The specific data analysis results are shown in Table 2.

According to further data analysis, after emphasizing the existence of morality, there was no significant difference in the sharing behaviors of participants toward human agents and robot agents (*F* = 1.000, *p* = 0.324). When the existence of morality is not emphasized, there is a significant difference in the sharing behaviors of participants toward human agents and robot agents (*F* = 7.062, *p* = 0.010, *d* = 1.181; for humans: *M* = 30.49, SD = 15.25; for robots: *M* = 10.61, SD = 18.27). The participants shared more with their human agents than with their robot agents. After emphasizing the possible impact of reputation, there was no significant difference in the sharing behaviors between human agents and robot agents among participants (*F* = 0.000, *p* = 1.000). When the potential impact of reputation is not emphasized, there is a significant difference in sharing behaviors between human agents and robot agents among participants (*F* = 3.981, *p* = 0.050, *d* = 1.245; for humans: *M* = 31.24, SD = 14.79; for robots: *M* = 12.34, SD = 15.56). The participants shared more with their human agents than with their robot agents. These data analysis results further prove that in human teams, humans naturally consider moral constraints and reputation constraints. In addition, they adjust their behavior accordingly, and this spontaneous consideration does not occur in human-robot teams. However, when moral constraints and reputation constraints are emphasized, the binding effects of morality and reputation on behavior are similar in both human and human-robot teams.

Overall, the results of Experiment 2 show that moral constraints and reputation constraints affect sharing behaviors in human-robot teams. After emphasizing the existence of morality and reputation in the team, participants will adjust their sharing behaviors with robot partners based on the impact of morality and reputation, and these sharing behaviors are similar to those in human teams. If there is no deliberate explanation of the possible impact of morality and reputation in the team, participants will not consider these two aspects independently and adjust their behavior accordingly, as in human society. This is consistent with the research results of Kahn et al. (2012), which suggest that humans apply different social rules to their human and robot partners, with the former spontaneously applying morality and reputation, while the latter does not. However, if it is explained to human-robot teams that morality and reputation are applicable team interaction rules, humans will adjust their own behavior based on these two rules, as in human society.

## 4 Experiment 3

### 4.1 Experimental design

#### 4.1.1 Participants

A total of 128 participants were recruited in this experiment, including 78 women and 50 men, with an average age of 20.66 years (SD = 1.86). The subjects were randomly divided into four groups, each with 32 people. The four groups were named the morality human group, morality robot group, immorality human group, and immorality robot group. There was no significant difference in the sex ratio or age among the four groups (both *p* > 0.05).

#### 4.1.2 Task and experimental design

The experiment adopted a 2 × 2 design (moral constraints: morality and immorality; agent type: human and robot). A dictator experimental paradigm similar to Experiment 1 and Experiment 2 was adopted. The experimental task is a round of independent dictator decision-making trials, with each participant experiencing only one type of agent (human or robot), that is, only a human-human team or human-robot team, and only one type of moral level (morality and immorality). For the morality human group, participants were informed that sharing behaviors might cause the human agent to go bankrupt. For the morality robot group, participants were informed that sharing behaviors may cause the robot agent to be scrapped. For the immorality human group and immorality robot group, Experiment 3 does not add similar experimental settings, and the experimental conditions are the same as Experiment 1, which does not emphasize moral constraints. The experimental settings for each group are shown in Table 3.

**Table 3:** Trial treatment.

Variables		Moral constraint	
		Morality	Immorality
Agent type	Human	Morality human group	Immorality human group
	Robot	Morality robot group	Immorality robot group

The test includes an independent variable of moral constraint (between-group variable, including two levels: morality and immorality), a moderating variable of agent type (between-group variable, including two levels: human and robot), an intermediary variable of reputation concern, and a dependent variable (sharing behaviors).

Among them, the independent variable, moral constraint, is controlled by whether the participant is informed before the experiment that their sharing behaviors will cause the participant’s human agent to go bankrupt or the robot agent to be scrapped, similar to the setting in Experiment 2. Specifically, for the morality human group, before the trial, participants were told that if the amount shared was too small, the other person (the human agent) would be penniless and their lives would become even more difficult. For the morality robot group, before the trial, the participants were informed that if the sharing amount was too small, the other party (the robot agent) would be penniless and unable to receive subsequent maintenance and would be scrapped. For the immorality human group and immorality robot group, the trial did not emphasize the consequences of participants’ sharing behaviors. To determine the experimental setup of the group in which the participants truly felt for the robot, after being informed of the corresponding moral constraint and agent type settings of the participants in the group, the participants needed to fill out the perceived moral scale. The questionnaire and measurement method used are consistent with Experiment 2.

#### 4.1.3 Measure

The regulating variable agent type was controlled through the 10-s video and 10-picture selection method.

The mediating variable reputation concern was measured using the same modified reputation scale developed by Zinko et al. (2016) as in Experiment 2.

The dependent variable, sharing behaviors, was measured using the number of cents the participants received in the experimental task.

In addition, the participants’ self-reported risk preference and perceived intelligence were measured as control variables, and the questionnaire and measurement method used were consistent with Experiment 1.

#### 4.1.4 Procedure

The experiment lasted approximately 15 min in total. Before the experiment began, the participants signed an informed consent form, read the experiment instructions, and completed the pre-experiment questionnaire, including gender, age, and risk preference. According to the requirements of the group, the subjects completed a 10-s video and 10-picture selection, and the participants were informed of the moral rules (whether there were moral constraints) and filled out the perceived moral scale. Then, the participants completed a round of dictator decision-making trials. Finally, the participants completed the post-experiment questionnaire, including the perceived intelligence scale. The research was approved by the ethics committee of the university where it is located.

### 4.2 Data analysis and results

According to the independent sample *t* test results, the perceived moral level of the morality human group (*M* = 3.92, SD = 0.42) was significantly higher than that of the immorality human group (*M* = 3.37, SD = 0.51), with *t* = 1.168, *p* < 0.001, *d* = 0.172. The perceived moral level of the morality robot group (*M* = 3.94, SD = 0.48) was significantly higher than that of the immorality robot group (*M* = 3.36, SD = 0.39), with *t* = 0.628, *p* < 0.001, *d* = 1.324. This indicates that the experimental setup is effective, and each group of participants has correctly perceived the level of moral constraints in their group’s experimental setup, allowing for further data analysis.

According to descriptive statistical analysis, in the absence of moral constraints, when confronted with human agents (*M* = 34.69, SD = 16.25), more money is shared than when confronted with robot agents (*M* = 1.91, SD = 3.47). Compared to the situation without moral constraints, in the situation with moral constraints, there is not much difference in the amount of money shared with human agents (*M* = 37.94, SD = 30.59), but the amount of money shared with robot agents (*M* = 34.38, SD = 19.68) increases numerically. In the absence of moral constraints, the level of reputation concern when confronted with human agents (*M* = 3.63, SD = 0.34) is higher than when confronted with robot agents (*M* = 2.64, SD = 0.47). Compared to the situation without moral constraints, in the context of moral constraints, the level of reputation concern in both human agents (*M* = 4.12, SD = 0.50) and robot agents (*M* = 3.78, SD = 0.48) has been improved. The specific results are shown in Table 4.

**Table 4:** Descriptive statistics of four trials.

		Reputation concern		Sharing behaviors	
		Mean	SD	Mean	SD
Immorality	Human	3.63	0.34	34.69	16.25
	Robot	2.64	0.47	1.91	3.47
Morality	Human	4.12	0.50	37.94	30.59
	Robot	3.78	0.48	34.38	19.68

The mediating effect of reputation concerns was tested. According to the results of the regression analysis, moral constraints have a significant positive impact on sharing behaviors (Model 1: *B* = 18.297, *p* < 0.001) and have a significant positive impact on reputation concern (Model 2: *B* = 3.316, *p* < 0.001). After considering the mediating variable of reputation concern, the impact of moral constraint on sharing behaviors is not significant (Model 3: *B* = 1.457, *p* = 0.738), while reputation concern has a significant positive impact on sharing behaviors (Model 3: *B* = 20.153, *p* < 0.001), which suggests that perceived reputation has a full mediating effect between the role of morality and sharing behaviors. According to the results, it is assumed that Hypothesis 3 is verified. The detailed analysis results are shown in Table 5.

**Table 5:** Results on the impact of morality constraints on sharing behaviors with the mediating role of reputation concerns.

	Model 1	Model 2	Model 3	Model 4	Model 5
	Sharing behaviors	Reputation concern	Sharing behaviors	Reputation concern	Sharing behaviors
Moral constraint	18.297**	3.316**	1.457	3.25**	−3.147
Reputation concern			20.153**		13.104**
Agent type				−32.781**	−19.742*
Moral constraint*agent type				29.219**	20.680*
R2	0.130	0.326	0.356	0.598	0.407
Adjusted R2	0.123	0.320	0.345	0.589	0.388
F	18.756	60.896	34.505	61.565	21.139

*Indicates that the figure is significant at the 0.5 level, ** indicates that the figure is significant at the 0.01 level.

The moderating effect of agent type on the impact of moral constraints on reputation concerns was tested. According to the results of the regression analysis, moral constraints have a significant impact on reputation concerns (Model 4: *B* = 3.25, *p* < 0.001), and the interaction between moral constraint and agent type also has a significant impact on reputation concern (Model 4: *B* = 29.219, *p* < 0.001), indicating that agent type plays a moderating role in the impact of moral constraints on reputation concerns. According to the results, it is assumed that Hypothesis 4a is verified. The detailed analysis results are shown in Table 5.

The moderating effect of agent type on the impact of moral constraints on sharing behaviors was tested. According to the results of the regression analysis, the impact of moral constraints on sharing behaviors is not significant (Model 5: *B* = −3.14, *p* = 0.547), while the interaction between moral constraints and agent type has a significant impact on sharing behaviors (Model 5: *B* = 20.680, *p* = 0.006), indicating that object types play a moderating role in the impact of moral constraints on sharing behaviors. According to the results, Hypothesis 4b is verified. The detailed analysis results are shown in Table 5.

**Figure 3:**
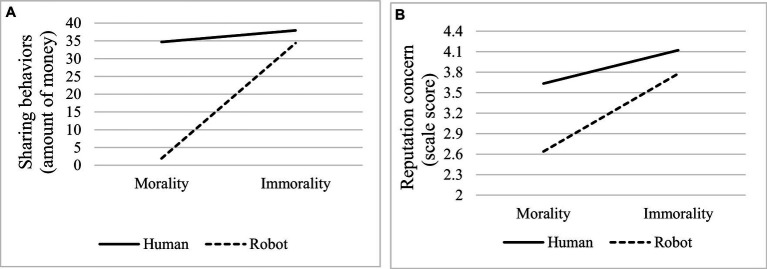
Simple slope test diagram. **(A)** A simple slope test of the moderating role of agent type in the influence of moral constraint on sharing behaviors. **(B)** A simple slope test of the moderating role of agent type in the influence of moral constraint on reputation concern.

Robustness testing on the regulatory effect of agent types was performed using simple effects analysis. For the moderating effect of agent type between moral constraint and sharing behaviors, when the agent is human, moral constraint has no significant impact on sharing behaviors (*t* = 0.522, *p* = 0.603, simple slope = 3.250); when the agent is a robot, moral constraint has a significant impact on sharing behaviors (*t* = 9.049, *p* < 0.001, simple slope = 32.649). The Chow test shows that the *F* value is greater than the critical value, and there is a significant difference between the two slopes (*F* = 13.826 > *F* (3,122) = 2.679). For the moderating effect of agent types between moral constraints and reputation concerns, when the agent is human, moral constraints have a significant impact on reputation concerns (*t* = 4.501, *p* < 0.001, simple slope = 0.488), but the impact is relatively small. When the agent is a robot, moral constraint has a greater and significant impact on reputation concern (*t* = 9.438, *p* < 0.001, simple slope = 1.140). The Chow test shows that the F value is greater than the critical value, and there is a significant difference between the two slopes (*F* = 27.586 > F (3,122) = 2.679). These conclusions all indicate that the regulatory effect of agent types has strong robustness. The slides are displayed in Figure 3.

Further analysis will be conducted on the moderating effect of agent type, exploring the proportion of direct and indirect influence paths of independent variables on dependent variables at different variable levels. For the moderating effect of agent type on moral constraint and sharing behaviors, when the agent type is human, according to bootstrap analysis, the direct impact path of moral constraints on sharing behaviors is not significant (effect = −3.121, 95%CI = [−13.447, 7.205]), but the indirect impact path of reputation concern is significant (effect = 6.371, 95%CI = [1.799, 12.115]), which indicates that in this case, the impact of moral constraint on sharing behaviors is entirely achieved through indirect pathways, and perceived reputation is a complete mediator. When the agent type is a robot, the direct impact path of moral constraints on sharing behaviors is significant (effect = 17.603, 95%CI = [4.679, 30.527]), and the indirect impact path of reputation concerns is significant (effect = 14.865, 95%CI = [5.403, 25.077]). The former has an effect of 54.22%, while the latter has an effect of 45.78%. This indicates that in this case, the impact of moral constraints on sharing behaviors is 54.22% through direct paths and 45.78% through indirect paths, and reputation concern is a partial intermediary. The detailed analysis results are shown in Table 6.

**Table 6:** The mediating effect results under two different agents.

Agent type		Effect	BootSE	95% CI
Human	Direct effect	−3.121	5.216	[−13.447, 7.205]
	Indirect effect	6.371	2.656	[1.799, 12.115]
Robot	Direct effect	17.603	6.529	[4.679, 30.527]
	Indirect effect	14.865	4.995	[5.403, 25.077]

## 5 Discussion

### 5.1 Discussion of experiment 1

Hypothesis 1 is verified. According to the results of Experiment 1, there are differences in sharing behaviors between human and robot partners. Humans are more willing to share with human partners, which is consistent with the research results of Sandoval et al. (2016), where participants provide much less money to robots than to human agents. Due to the excessive similarity between robots and humans, human beings as a group have concerns about robots, as this similarity blurs the boundaries of categories and undermines human uniqueness (Ferrari et al., 2016). People have the motivation to view their own group as different from others (Huang et al., 2021). However, even though there is currently a tendency toward anthropomorphic robots, it is difficult for humans to place robots and humans in the same position when sharing. Compared to sharing with robots, humans apply more social norms when sharing with other humans (Dehghani et al., 2010).

### 5.2 Discussion of experiment 2

Hypothesis 2 is verified. According to the results of Experiment 2, moral constraints and reputation constraints, that is, considering moral rules and reputation, will affect sharing behaviors in human-robot teams. Previous studies have shown that social norms affect human sharing behaviors (Wei et al., 2023), with morality and reputation being two important foundation. Sharing behaviors that conform to social norms is crucial for establishing and maintaining social relationships (Strauß and Bondü, 2023).

The differences in sharing behaviors between human-robot teams and human teams may stem from considerations of moral rules. When there are no clear moral rules, there are differences in how humans share with their human and robot partners. In human teams, even without emphasizing clear moral rules, people tend to share their resources with other human partners (Kogut, 2012). However, in human-robot teams, even if robots are considered to have emotional experiences or needs similar to those of humans, there are still significant differences between the two when moral rules are not emphasized (Nijssen et al., 2021b). Compared to human partners, the moral norms imposed on robot partners are different (Malle et al., 2015; Giroux et al., 2022). When sharing with human partners, people tend to consider morality more (Dehghani et al., 2010), believing that they need to bear more moral responsibility for other humans than for robots (Saygin et al., 2012). These results indicate that when there is no emphasis on moral rules; when dealing with human partners, humans will naturally consider moral rules and share more resources. When dealing with robot partners, they will not consider moral rules as they do with human partners and share fewer resources.

Previous studies have shown that people are not completely unwilling to share money with robots (Heijnen et al., 2019). Robots are becoming increasingly similar to humans, leading us to view them as more than just tools. The psychological mechanisms and processes when humans interact with robots are similar to those when interacting with other humans. When clarifying moral rules, people project human psychological characteristics onto robots (Nicolas and Agnieszka, 2021), which are endowed with emotional richness and depicted as an emotional entity and are endowed with thoughts similar to humans, applying human social rules when interacting with them. When confronted with robot partners, they will share resources as if they were human partners. When a robot is subjected to physical abuse, people will sympathize with the robot. In situations where the life of a robot is threatened, humans may even sacrifice a group of anonymous humans to save the robot’s “life” (Nijssen et al., 2021b).

The differences in sharing behaviors between human-robot teams and human teams may also stem from considerations of reputation impact. On the one hand, gaining reputation to a certain extent reflects an individual’s dependence on partners (Sperber and Baumard, 2012). Individuals hope to establish an expectation through interaction with their partners that they will contribute to their goals and expected states, thereby becoming dependent on their partners. In human teams, this dependence naturally exists, and people are highly concerned about reputation (Kupfer and Giner-Sorolla, 2021). Reputation considerations in interactions have always existed. When an individual’s selfish desires conflict with the needs or social norms of others, to gain a higher reputation, they will be given more resources during the sharing process (Sperber and Baumard, 2012). Even when the interaction rules are not clear, individuals may exhibit certain sharing behaviors due to considerations of reputation impact.

In human-robot teams, human perception of robots has a significant impact on interdependence. This perception involves not only the comfort and acceptability of human interaction with robots but also the potential activation of basic human reactions during interaction with robots, such as the attribution of thinking and moral abilities. Due to the simplicity of the existing robot products and the small impact of the relevant market, people have not yet established a deep perception of robots, so there is no natural dependence on robots. Only when the interaction rules clearly define the reputation factors can people consider the evaluation of robots on their own reputation. In addition, humans apply psychological mechanisms similar to those of humans when thinking about and evaluating robots (Lamba and Mace, 2010). For example, when playing games with robot opponents, people may use strategies similar to those of humans during wartime (Torta et al., 2013). Therefore, when the interaction rules are clear, humans will consider the impact of the interaction behavior between humans and robots on their own reputation. However, compared to sharing with human partners, humans believe that robots do not make decisions that affect their own well-being and are insufficient to damage their reputation. Therefore, when reputation factors are not clearly defined in the interaction rules, reputation issues are not considered in the interaction behavior between humans and robots.

### 5.3 Discussion of experiment 3

Hypothesis 3 in Experiment 3 has been validated, indicating that reputation concern plays a mediating role between moral constraint and sharing behaviors. Morality ensures a good reputation (Sperber and Baumard, 2012), and the pursuit of reputation itself is an adaptation to morality (Nordin, 2015). Moral constraints make people pay more attention to reputation, leading to the sharing of more resources. In economic games, strengthening moral constraints increases the importance of reputation, and individuals exhibit more cooperative tendencies (Michael and Salice, 2017).

Moral constraints are mainly based on self-interest and are related to good reputation considerations (Hanoch et al., 2021). Morality originates from actively establishing and maintaining a person’s reputation; that is, the purpose of morality is to enable an individual to gain a good reputation. From the perspective of reciprocity, the role of morality is to enhance an individual’s reputation and make them reliable partners in the team (Nijssen et al., 2021a), which means that ethical behavior is actually a self-serving way to gain a good reputation, especially if a person’s reputation is taken into account (Haley and Fessler, 2005). However, if people only consider gaining a good reputation, they may not be trusted due to selfish motives, and from a reciprocal perspective, they may be rejected as partners. From a long-term perspective, a reputation lacking morality may be difficult to maintain (Sperber and Baumard, 2012).

The moral behavior of individuals, including sharing behaviors, is clearly governed by their concern for reputation. The biological function of this type of behavior is to help individuals gain a good reputation as sharers (Sperber and Baumard, 2012; Torta et al., 2013; Schier et al., 2016). People may control selfish behavior and exhibit more sharing behaviors because they intrinsically value doing so—a genuine moral reason—or gaining the approval of others—an instrumental reason. Especially when there are signs that behavior may be observed and considered objectionable, individuals are more likely to give up short-term gains and consciously or unconsciously pursue a better reputation, thereby sharing more resources; that is, superficial sharing behaviors are actually dominated by reputation attention. Even in anonymous situations, individuals may still be affected by concerns about their reputation, showing generosity and sharing resources (Clarkson, 2022). The focus on reputation promotes sharing behaviors in economic games (Piazza and Bering, 2008). Sharing behaviors are actually guided to some extent by self-interest, and the self-interest motivation to maintain reputation may be sufficient to explain cooperative behavior in the absence of direct returns (Nordin, 2015).

Hypothesis 4a is verified, and the agent type has a moderating effect between moral constraints and reputation concerns; that is, when humans are confronted with humans, there is a difference in the impact of moral constraints on the level of reputation concern. However, when humans are confronted with robots, there is a more significant difference in the level of reputation concern with or without moral constraints. Moral constraints usually affect reputation concerns (Lee et al., 2021), and considering moral rules can increase the importance attached to reputation. In human teams, moral norms instinctively lead to consideration and attention to reputation. After emphasizing moral norms, reputation concerns will naturally increase, but individuals will naturally consider moral norms when confronted with human partners, so emphasizing moral norms has a smaller impact on reputation considerations. The morality applied to robots in human-robot teams still differs from that in human teams (Seibt et al., 2016). However, by emphasizing moral norms within the team, robots are more likely to be endowed with anthropomorphic tendencies, and this emphasis on moral norms naturally reinforces individuals’ consideration of robots’ own reputation evaluation.

Hypothesis 4b is verified, and the agent type has a moderating effect between moral constraints and sharing behaviors. When confronted with human partners, there is no significant difference in sharing behaviors with or without moral constraints, but when confronted with robot partners, there is a significant difference in sharing behaviors with or without moral constraints. Previous studies have shown a strong relationship between morality and sharing behaviors in dictator games (Schier et al., 2016). When confronted with human agents, moral norms are naturally considered, and even if moral rules are not emphasized, people tend to share their resources with other human partners (Sperber and Baumard, 2012). Emphasizing morality does not have a significant impact on sharing behaviors. However, when confronted with robot agents, without emphasizing social rules, humans are inclined to believe that robots have no emotional experiences or needs similar to humans, and robots will not be fully integrated in the interaction process and human social moral norms, and the resources shared are also limited. However, when moral rules are clarified, robots are endowed with a stronger tendency to personify, given more moral attention, integrated with similar concepts, processes, and authorizations (Ogunfowora et al., 2021) when interacting with humans, and more resources are shared with them.

## 6 Limitations and future work

We recognize that our research still has certain limitations. First, this study draws on the paradigm of economic game experiments, where participants’ sharing behaviors are related to money. In addition to money-related sharing, the content of sharing can also include knowledge, information, experience, stress, negative emotions, etc. Some studies suggest that there may be differences in sharing behaviors and their impact on other factors when sharing content is different (Voiklis et al., 2016). Therefore, future research can further explore the differences between human-robot teams and human teams in other types of sharing behaviors. Second, the sharing behaviors conducted in this study use the dictator game paradigm, and the participants cannot provide any feedback on the participants’ decisions. Feedback and communication between the sharer and the one being shared with, as well as other experimental limitations, may have an impact on sharing behaviors. Therefore, future research can further draw on other types of experimental paradigms to explore sharing behaviors in human-robot teams more extensively. Third, the object of this study is a “1 V1” interactive human-robot team, but currently, HRI also includes more complex scenarios. Future scenarios are no longer limited to single robots operating in various human environments, such as single human individuals interacting with multiple robots, multiple human individuals interacting with single robots, and interactions between multiple robots and multiple human individuals. According to the theoretical hypothesis of social psychology, when people connect with individuals or groups, there are differences in their behavior (Balle, 2022); that is, there are differences in human behavior in teams of different sizes and structures. Therefore, future research can explore the differences in sharing behaviors among different team sizes (two or more people) and team structures (proportion of robots).

Fourth, this study explores the impact and mechanisms of moral constraints and reputation constraints on sharing behavior. The mechanisms that influence sharing behavior and moral decision-making are relatively complex, and there are other social factors such as reciprocity, fairness, altruism, moralism, utilitarianism, and free riding that may also affect sharing behavior. Empathy also has an impact on moral decision-making. Therefore, Future research will further explore the impact mechanisms of these social factors on human-robot team behavior. Fifth, the gender ratio of the participants in Experiment 1 of this study is imbalanced. Some studies have shown that in anonymous environments, women usually share more than men. Experiment 2 and Experiment 3 focus on gender balance, and in future more in-depth research, attention should be paid to the issue of gender balance. Sixth, the study informs participants that the money allocated is fictitious and unexpected rather than earned by themselves. Restrictions on sharing resources can affect participants’ sharing behavior. Some researchers have used the Ultimatum Game and Dictator Game experimental paradigms to convert the number of rewards obtained in the game into actual “take home” rewards. Therefore, Future research can further explore the impact of different restrictions on sharing resources on sharing behavior. Seventh, the participants of this study are undergraduate and graduate students. In the experiment, the identity of the robot fake participants faced by the participants is that of graduate students. Currently, it is not clear whether the identity of the robot fake subjects will affect the experimental mechanism, so more in-depth exploration can be conducted in the future. Finally, the participants of this study are students in China, and studies have shown that different cultural backgrounds can affect the way people share resources in psychoeconomic games. Therefore, future research can target participants from other cultural backgrounds and conduct more extensive research to obtain richer experimental results.

## 7 Conclusion

The role of robots in people’s work and life is increasingly prominent, and a better understanding of the interaction mechanism between humans and robots is crucial, which can help predict and judge the internal laws of interaction and work achievement performance in future human-robot collaborative teams. We not only revealed the differences in human sharing behaviors toward others and robot partners through three experiments that were conducted layer by layer but also proposed that the underlying mechanism behind these differences is the influence of moral constraints and reputation constraints and further revealed the impact mechanism of moral constraints and reputation considerations in human-robot teams. Specifically, Experiment 1 explored the sharing behaviors when confronted with human and robot partners, and the results showed significant differences in sharing behaviors between the two scenarios. Second, in Experiment 2, two conditions of moral constraints and reputation constraints were set to explore whether these two constraints are the mechanisms that cause differences. The results showed that in both human and human-robot teams, moral constraints and reputation constraints are the reasons for differences in sharing behaviors. Finally, Experiment 3 further explored the impact mechanism of moral constraints and reputation concerns on sharing behaviors. The results showed that reputation concern played a mediating role in the impact of moral constraint on sharing behaviors, and agent type played a moderating role in the impact of moral constraint on reputation concern and sharing behaviors.

The research results contribute to a better understanding of the interaction mechanism of human-robot teams. In future human-robot collaborative teams, basic moral rules can be formulated to reduce team friction and adaptability. In addition, the research results once again prove that moral constraints can increase people’s attention to reputation. In the construction of human-robot collaborative teams, people should be more aware of the importance of robot evaluation for their reputation. Finally, future human-robot collaboration team rule-making and interaction environment settings can consider the potential motivation of human behavior from both morality and reputation perspectives to achieve better work performance.

## Data availability statement

The raw data supporting the conclusions of this article will be made available by the authors, without undue reservation.

## Ethics statement

The studies involving humans were approved by Beijing University of Chemical Technology. The studies were conducted in accordance with the local legislation and institutional requirements. The participants provided their written informed consent to participate in this study. Written informed consent was obtained from the individual(s) for the publication of any potentially identifiable images or data included in this article.

## Author contributions

NC: Writing – original draft, Writing – review & editing, Data curation, Supervision, Validation, Conceptualization, Funding acquisition, Project administration, Resources, Software. XH: Writing – original draft, Writing – review & editing, Investigation, Methodology, Data curation, Formal analysis, Project administration. YZ: Writing – original draft, Writing – review & editing, Conceptualization, Formal analysis, Data curation, Investigation.
